# Use of Indocyanine Green Fluorescence in the Assessment of Bowel Perfusion in Emergency and Elective Colorectal Surgery: A Retrospective Cohort Study

**DOI:** 10.7759/cureus.117227

**Published:** 2026-09-30

**Authors:** Stefano Rossi, Biagio Picardi, Simone Rossi Del Monte, Francesco Cortese, Gennaro Mazzarella, Caterina Puccioni, Irnerio Angelo Muttillo

**Affiliations:** 1 Emergency Department, Ospedale San Filippo Neri, Rome, ITA; 2 Emergency Department, Sapienza University of Rome, Rome, ITA; 3 Emergency Department, Università Cattolica del Sacro Cuore, Rome, ITA

**Keywords:** anastomotic leakage, colorectal surgery, emergency, fluorescence, icg, indocyanine green (icg), laparoscopic

## Abstract

Background: The use of indocyanine green (ICG) fluorescence is a well-established technique in colorectal surgery for the evaluation of bowel stump perfusion. However, there is still no definitive acceptance, except for intraoperative macroscopic evidence, with reference to the incidence of anastomotic leakage (AL). The objective of this study is to evaluate whether ICG-guided fluorescence angiography reduces AL rates in both elective and emergency colorectal surgery.

Methods: This was a single-center retrospective comparative cohort study. From January 2019 to June 2020, adult patients undergoing elective or emergency colorectal surgery (right/left hemicolectomy, rectal resection, or Hartmann reversal) were enrolled (N=79). In the ICG group (N=40), ICG angiography was used to evaluate colonic stump perfusion before and after creating the anastomosis. Results were compared to a historical control group (N=39) operated without ICG guidance. Group comparisons were analyzed using Pearson's chi-square test.

Results: A total of 40 patients underwent surgery; 21 (52.50%) had benign pathology, and 19 (47.50%) exhibited malignant neoplasia. Thirteen (32.50%) were emergency surgeries, and 27 (67.50%) were planned elective surgeries. In almost all cases, the postoperative course was regular; in only one (2.5%) case of transanal total mesorectal excision (TaTME), there was an AL. Other complications were not related to the anastomosis, but some validated the excellent perfusion despite episodes of prolonged acute ischemia due to postoperative hemorrhage. Data were finally compared to a control group of 39 patients where the ICG fluorescence was not used.

Conclusions: The study demonstrates a positive association between the use of ICG fluorescence as a method for intraoperative assessment of bowel perfusion and its effectiveness even in emergency conditions and in acute postoperative hemorrhage, detecting an incidence of 2.5% (one case out of 40) of AL. In addition, ICG fluorescence led to a change in the surgical strategy during the procedure. It’s evident that to validate our results, further randomized studies on a larger data set are required. It would also be beneficial to evaluate the fluorescence between the mucous and serous layers quantitatively to confirm the reduction of AL rate, better evaluation of bowel perfusion, and, especially in emergency surgeries, the potential reduction of further operations.

## Introduction

Anastomotic leakage (AL) is the most feared complication in intestinal surgeries, particularly in colorectal surgeries [1]. The incidence varies between 1.2% and 19.2% for all interventions, with a peak of 28% in rectal cancer with low or ultra-low localization without a defunctioning stoma [2], with a higher incidence and variability in emergency surgery cases [1-3]. Despite the important technological advances of the last few years in terms of technique and surgical devices, AL complications have always remained significant and unchanged in terms of incidence, with inevitable prolongation of hospital stay and costs. In 95% of cases, the AL required further surgery with a high mortality rate [1,4]. Furthermore, more than half of the patients requiring re-operation will have a permanent stoma [1] as a consequence of this further surgery. Although the etiopathogenesis involves several factors, the main and most determining factor is the adequate evaluation of the vascular supply to the intestinal tissues [3-5]. To date, the assessment of good vascular supply has been evaluated only using clinical criteria (color of the tissues, bleeding of the edges, and arterial pulse). These clinical criteria are insufficient to make objective and quantifiable judgments during surgery. The angiographic visualization with fluorescence, by means of a dedicated near-infrared (NIR) endoscopic video camera, allows the surgeon to view in NIR light the presence in the tissues of a fluorescence dye, indocyanine green (ICG). ICG is administered intravenously, which binds to plasma proteins and reaches all organs and regions of the body with excellent tolerability, few side effects, and extremely low toxicity, with the ability to absorb light in the NIR wavelength between 600 and 900 nm. The intensity of its visualization is proportional to its quantity in the tissues and, therefore, to the vascular perfusion of the same. While ICG has been studied extensively in elective settings, its role in emergency colorectal surgery, where vascular compromise and patient instability are more common, remains less explored. Therefore, this study aims to evaluate ICG's utility in both elective and emergency contexts, evaluating the impact of this intraoperative imaging technique on the perfusion of the intestinal stump to obtain an optimal anastomosis, reduce AL, decrease re-operations, and increase the number of primary anastomoses. We hypothesized that ICG-guided perfusion assessment reduces anastomotic leakage rates compared to standard clinical visual assessment alone in both elective and emergency colorectal surgery.

This article was previously posted to the Research Square preprint server on October 12th, 2021 [6].

## Materials and methods

This was a single-center retrospective observational study conducted at the Department of General and Emergency Surgery of San Filippo Neri Hospital, Rome, Italy, between January 2019 and June 2020. During the selected study period, we used fluorescence with ICG to evaluate the perfusion of the colonic stumps before and after the packaging of the anastomosis in emergency and elective colorectal surgery. A total of 79 patients undergoing elective or emergency colorectal surgery were enrolled using consecutive convenience sampling during this period. 

Inclusion criteria

The study included adult patients (≥18 years old) who underwent elective or emergency colorectal surgery with primary anastomosis or stump creation during the study period. 

Exclusion criteria

Patients with a history of severe allergic reactions to iodine or ICG, severe hepatic insufficiency, or refusal to consent were excluded.

Patients were divided into two operational cohorts. Thirty-nine patients were included in the control group, being operated on without using ICG to evaluate the perfusion of the colonic stump and of the anastomosis, due to several factors such as inexperience of the surgeon and inadequate setting or timing of surgery. Of these patients, 20 (51.28%) were operated for colonic malignancy and 19 (48.72%) for benign pathologies, 15 (38.46%) in an emergency setting, and 24 (61.54%) in an elective setting. Twenty-one patients (53.85%) had associated cardiovascular, pulmonary, or metabolic comorbidities, and seven patients (17.95%) had a BMI greater than 30. In parallel, a group of 40 patients, the ICG group, was evaluated with the ICG method; 21 (52.50%) were operated on for benign pathology and 19 (47.50%) for malignant neoplasia; 13 (32.50%) were in an emergency setting and 27 (67.50%) in an elective setting. Nineteen patients (47.5%) had associated cardiovascular, pulmonary, and metabolic comorbidities, and six (15%) had a BMI greater than 30 kg/m². Potential confounding variables, such as age, gender, BMI, severe comorbidities (hypertension, diabetes, ischemic heart disease), surgical urgency, underlying malignancy, surgical approach (laparoscopic vs. open), and adherence to Enhanced Recovery After Surgery (ERAS) pathways, were recorded (Table 1). Sample size was dictated by the total volume of eligible patients presenting to the department during the fixed study window (January 2019 to June 2020); no explicit preliminary sample size calculation was performed due to the exploratory nature of the study.

**Table 1:** Patients characteristics Values are expressed as mean (SD = standard deviation) or n (%). ICG: indocyanine green-guided colorectal surgery; BMI: body mass index; ERAS: Enhanced Recovery After Surgery

	ICG (40 patients)	Control (39 patients)
Age (years)		
Mean (SD)	62.6 (10.5)	67.74 (13.4)
<50 n (%)	3 (7.5)	1 (2.5)
50-70 n (%)	30 (75)	25 (64.2)
71-90 n (%)	7 (17.5)	13 (33.3)
Gender n (%)		
Female	23 (57.5)	20 (51.3)
Male	17 (42.5)	19 (48.7)
BMI kg/m^2^,n (%)		
<20	0 (0)	0 (0)
20-24	28 (70)	11 (28.2)
25-29	6 (15)	19 (48.7)
>30	6 (15)	9 (23.1)
Comorbidity n (%)		
Hypertension	15 (37.5)	11 (28.2)
Diabetes	7 (17.5)	3 (7.7)
Ischemic heart disease	3 (7.5)	3 (7.7)
Previous abdominal surgery	2 (5)	2 (5.1)
Psycosis	2 (5)	2 (5.1)
Smoking	2 (5)	3 (7.7)
Dyslipidemia	1 (2.5)	3 (7.7)
Colorectal disease n(%)		
Benign	21 (52.5)	19 (48.7)
Malignancy	19 (47.5)	20 (51.3)
Setting n (%)		
Urgency	13 (32.5)	15 (38.5)
Election	27 (67.5)	24 (61.5)
ERAS n (%)	26 (65)	4 (10.3)

Continuous variables were evaluated for normality using the Shapiro-Wilk test and are reported as mean ± standard deviation (SD). Categorical variables are presented as absolute numbers and percentages (n, %). Group comparisons were performed using the Pearson's chi-square test. Statistical significance was set at p<0.05. All statistical analyses were processed using IBM SPSS Statistics software, version 27 (IBM Corporation, Armonk, NY, USA).

ICG administration and imaging protocol

ICG powder (Verdye (5 mL/25 mg), Diagnostic Green GmbH, Kirchheim bei München, Germany) was diluted in 10 mL of sterile pyrogen-free distilled water (for injections) and administered intravenously in the patient’s peripheral vein, with the dose calculated per the patient's body weight. ICG was developed for photography by Kodak as early as 1955 and approved for clinical use in 1959 by the US FDA [7]. The molecule, when injected into the bloodstream, binds to plasma proteins (especially lipoproteins); it reaches arteries and veins in five to 50 seconds, and it is rapidly excreted by the liver in the bile, where it appears after about eight minutes, depending on the vascularization and liver function. Fluorescence remains for several hours. The standard dose for clinical use (0.1-0.5 mg/ml/kg) is well tolerated. The ICG gives off fluorescence when a laser or NIR light is excited at around 820 nm and is detected using dedicated cameras and optics (Figure 1).

**Figure 1:**
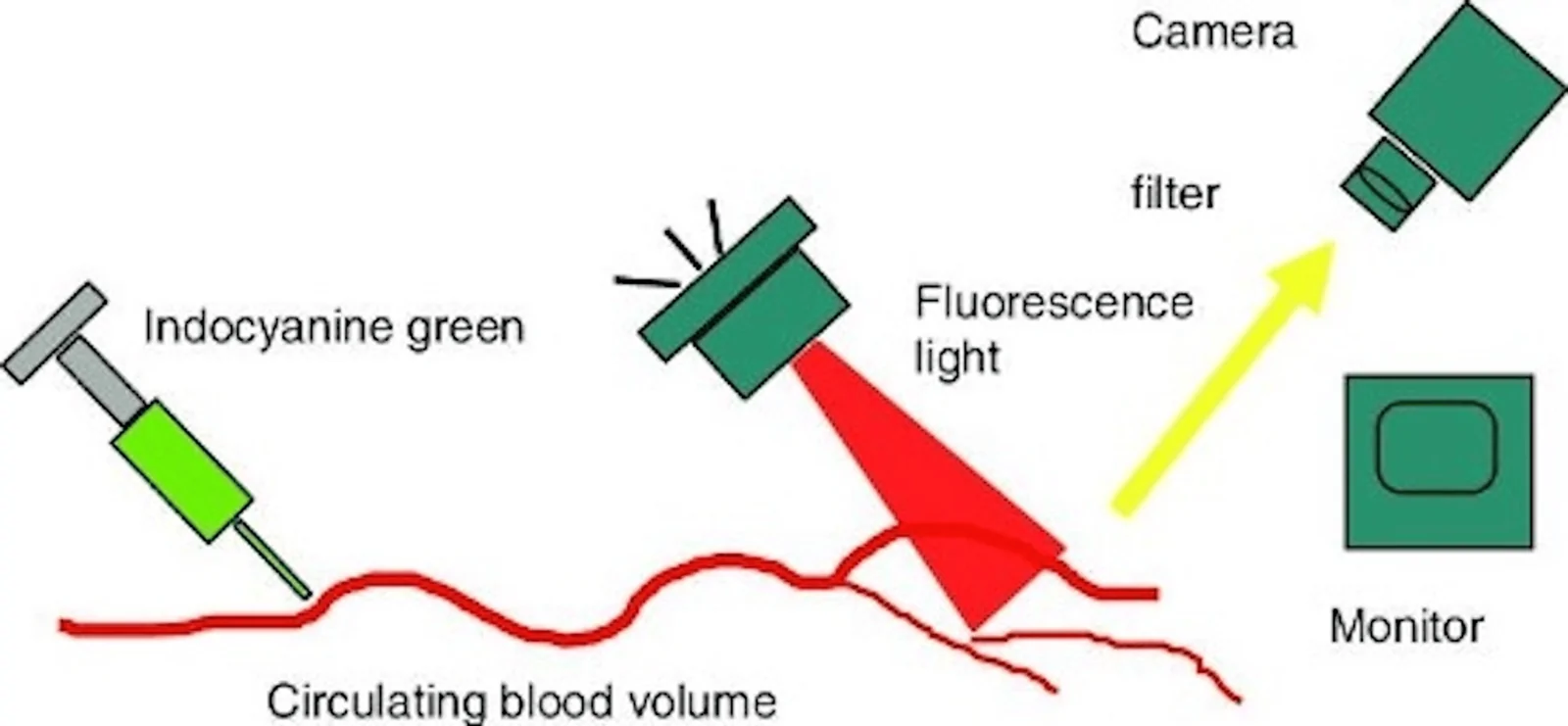
Indocyanine green near-infrared fluorescence This is an original image created by the authors using Microsoft PowerPoint (Microsoft Corporation, Redmond, WA, USA).

We used a dedicated laparoscopic system (KARL STORZ GmbH & Co. KG, Tuttlingen, Germany) with the image generated by a full HD camera (IMAGE 1 SPIESTM, KARL STORZ GmbH & Co. KG, Tuttlingen, Germany) connected to a laparoscope at 30 degrees and 10 mm in diameter with a specific filter for NIR fluorescence. The light source (D-LIGHT P SCB, KARL STORZ GmbH & Co. KG, Tuttlingen, Germany) then allows, by the use of a foot pedal, to "switch" between NIR and standard light according to the surgeon's needs. In the case of intestinal perfusion, in particular, the epiploic appendages and the mesentery are "colored" first, then it reaches the intestinal wall (the antimesenteric margin of the descending and transverse colon is paler due to the greater thickness), and, if well vascularized, the wall gradually turns a bright green. The dose we used was 0.3-0.4 mg/ml/kg in a single administration before the resection of the proximal colonic stump or administered twice (in a dose of 5 mL each) before the section of the stump and after the packaging of the anastomosis. Given the rapid appearance of the dye in the bloodstream, the timing of administration is practically instantaneous (about 50 seconds before the evaluation of perfusion). In case of anterior rectal resection, we also carried out endoluminal control of the anastomosis. The surgical techniques performed are described in Table 2.

**Table 2:** Surgical procedures Values are expressed as n (%). ICG-CRS: indocyanine green-guided colorectal surgery; TaTME: transanal total mesorectal excision; ICG: indocyanine green; Redo: reanastomosis

	ICG-CRS (40 patients)	Control (39 patients)
Type of surgery n (%)		
Right hemicolectomy	7 (17.5)	22 (56.4)
Left hemicolectomy	14 (35)	9 (23.1)
Transverse colectomy	2 (5)	1 (2.6)
Rectal anterior resection	3 (7.5)	0 (0)
TaTME	4 (10)	0 (0)
Atypical resection	2 (5)	0 (0)
Hartmann’s reversal	8 (20)	6 (15.4)
Subtotal Colectomy	0 (0)	1 (2.6)
Technique n (%)		
Laparoscopic	31 (77.5)	4 (10.3)
Open	9 (22.5)	35 (89.7)
Anastomosis ICG review n (%)		
Redo	0 (0)	0 (0)
Revision of strategy	3 (7.5)	0 (0)

Furthermore, in the last 18 patients operated in order of time (seven left hemicolectomies, five right hemicolectomies, one transanal total mesorectal excision (TaTME), three anterior rectal resection, two Hartmann reversal) we carried out a double pre-anastomotic evaluation of the serous (Figure 2) and mucous side (Figures 3A, 3B) simultaneously (then also evaluated by post-anastomotic endoluminal view only the rectum), in consideration of the different sensitivity to ischemia of the two wall layers to of the mucous one, as evidenced by recent studies [8]. 

**Figure 2:**
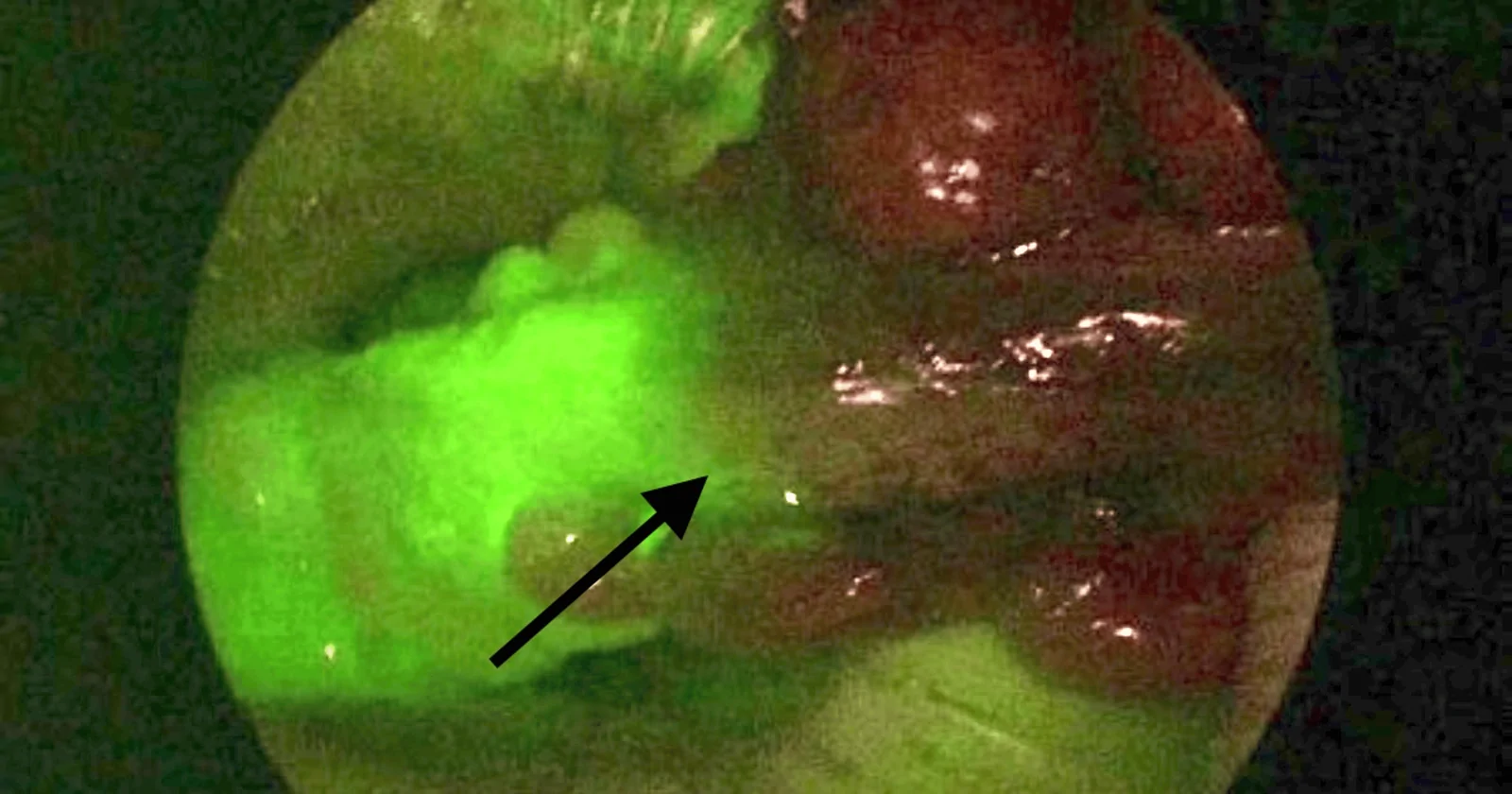
Indocyanine green (ICG) fluorescence-guided bowel perfusion assessment prior to colon resection; the black arrow indicates colonic perfusion demarcation with ICG.

**Figure 3:**
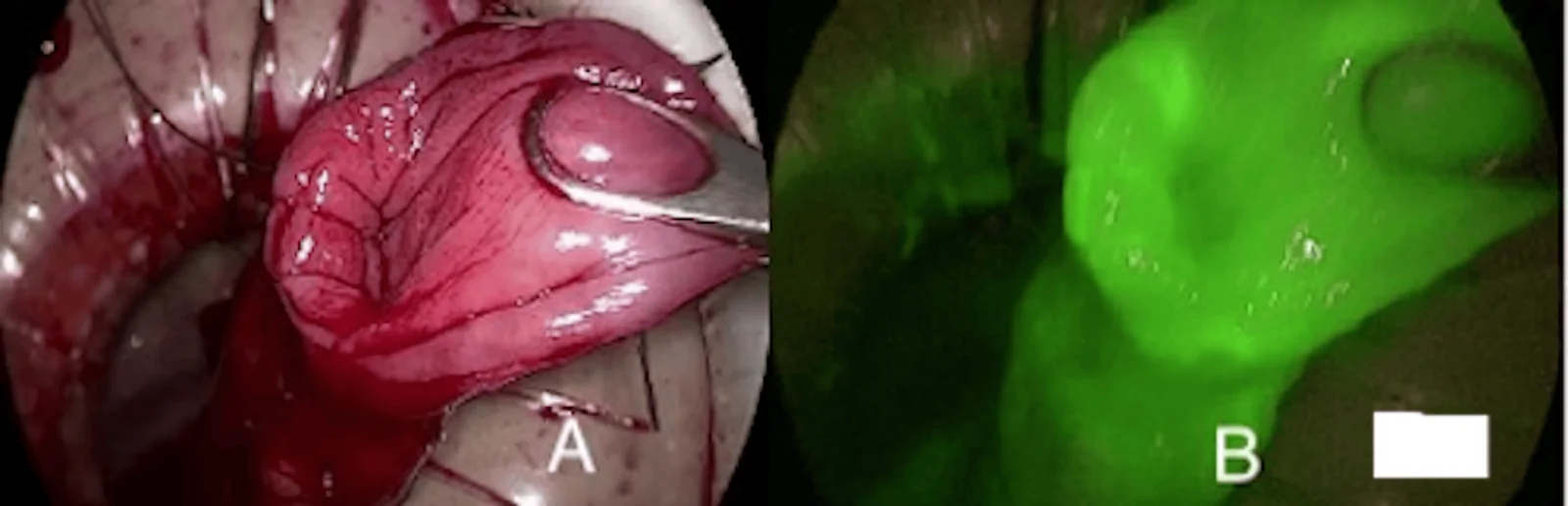
Assessment of bowel mucosal perfusion using indocyanine green (ICG). (A) Pre-anastomotic check of the mucosal side with standard light. (B) Pre-anastomotic check of the mucosal side with ICG fluorescence.

Due to the technological system in those cases operated in laparoscopy, in which the colic stump was prepared extracorporeally, and in all open operations, in order to clearly visualize the fluorescence and its intensity in the different colic segments, the chamber of the operating theatre must be darkened with lights reduced, with the visual field reduced in diameter as necessary. This technological limit will be overcome with the imminent introduction of real-time unification of fluorescence images with infrared (IR) and simultaneous white light (real-time overlay).

Ethics

All procedures performed in this study were in accordance with the Declaration of Helsinki and with the ethical standards of the hospital research committee that approved the study. All experimental protocols and all methods were carried out in accordance with relevant guidelines and regulations. All participants signed informed consent prior to enrolling in this study.

## Results

In general, surgical procedures guided by fluorescence with ICG have demonstrated advantages in the patient’s length of stay (LOS) in hospital, especially for colorectal surgery, with a reduction in AL rates and reanastomosis (redo), at the expense of a slight increase in operating time. This study, as well as multiple studies completed to date, confirms that this imaging technology is safe, effective, and sustainable [9]. In the control group, a total of 39 patients (19 males and 20 females), with an average age of 67.7 ± 13.4 years, underwent colorectal surgery with primary anastomosis, 19 for benign pathologies and 20 for malignancy.

We performed nine left colectomies (two laparoscopic and seven open, one with ileus resection associated and one with splenectomy associated), 22 right colectomies (one laparoscopic, one with duodenal resection associated, one with ureteral resection associated, one with jejunal and bladder resection associated), one transverse resection, six Hartmann reversals (one laparoscopic), and one subtotal colectomy. In the ICG group, a total of 40 patients (17 males and 23 females), with an average age of 62.6 ± 10.5, underwent ICG-guided colorectal surgery, 21 for benign pathology and 19 for malignant neoplasia. Of these, 13 operations were carried out in an emergency setting and 27 as elective procedures (Table 1). In this group, we performed seven right hemicolectomies (two open and five laparoscopic), 14 laparoscopic left hemicolectomies, two laparoscopic transverse resections, three laparoscopic anterior rectal resections, four TaTME with protective ileostomy, eight Hartmann reversals (six open and two laparoscopic), one laparoscopic resection and anastomosis for colo-cutaneous stenosis and fistula after left hemicolectomy for neoplasia, and one open resection and anastomosis for stenosis after left hemicolectomy for diverticulitis with ureteral reconstruction (Table 2). In all the cases of malignant neoplasia, we followed a classic medium-lateral approach with "high" vascular ligation. In cases of benign pathology and for Hartmann’s reversal procedures, we have opted, in most cases, for peripheral vascular ligatures, especially in the case of elderly patients with significant associated comorbidities. The same strategy was used in emergency cases. The evaluation of the ICG perfusion was carried out before the resection of the proximal stump on the serous and mucous side (Figures 2, 3) and after the packaging of the anastomosis on the serous side (Figures 4A, 4B), on the mucous side only in the case of rectal resection. In fact, in the case of "low" rectal resection, the fluorescence evaluation of the distal stump perfusion was difficult to visualize on the serous side for anatomical reasons (distal part of the anorectal canal) and was almost exclusively entrusted to the evaluation of the mucous side by endoluminal view (Figure 5). No adverse reactions were recorded during the administration of ICG. We did not perform a redo in the treated patients, but we did a revision of the section line (revision of decision) of the proximal ileal stump in a right hemicolectomy, in a Hartmann’s reversal, and in a left hemicolectomy (7.5%). In cases where we carried out a strategy review, we found no AL nor any other complication. 

**Figure 4:**
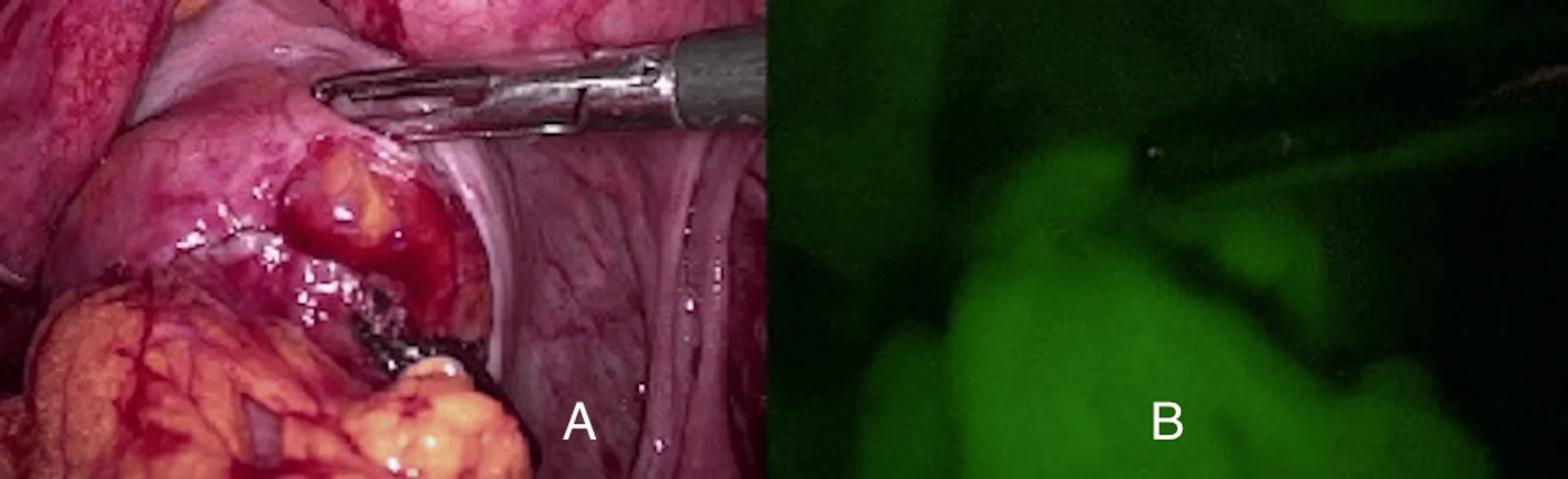
Bowel serosal indocyanine green (ICG) perfusion assessment after packaging of the anastomosis. (A) Post-anastomotic check of the serous side with standard light. (B) Post-anastomotic check of the serous side with ICG fluorescence.

**Figure 5:**
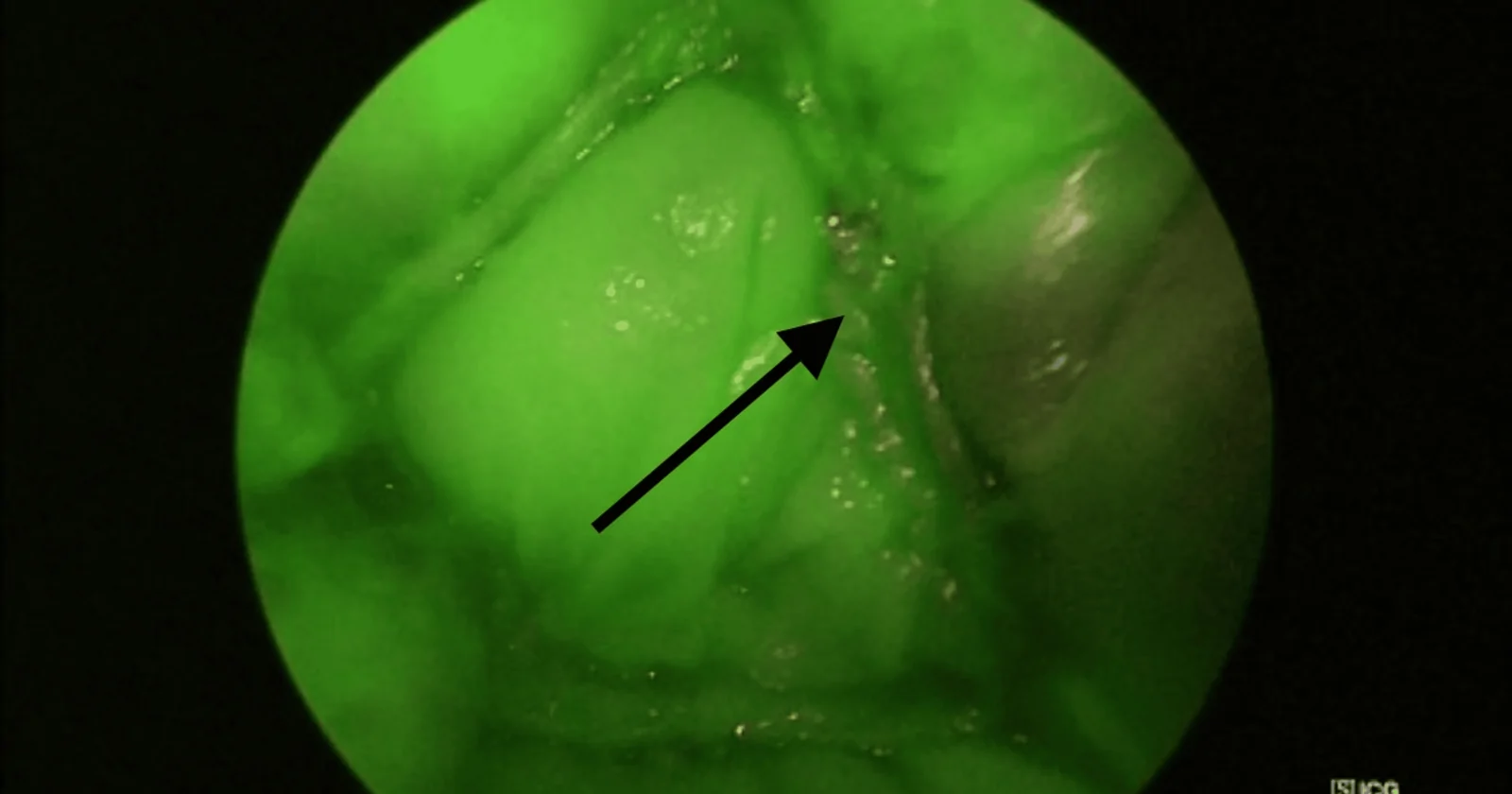
Endoluminal transanal indocyanine green perfusion assessment of the mucosal side after colorectal anastomosis. The black arrow indicates the colorectal anastomotic line.

The postoperative course in the control group was characterized by a morbidity rate of 41.03%: there were five grade I complications according to the Clavien-Dindo classification [10], four grade II complications, two grade IIIa complications, two grade IIIb complications, and five grade V complications. Among anastomotic complications, we registered seven cases of AL (17.95%); two of them underwent relaparotomy but died despite resurgery because of multi-organ failure (MOF) and two others died without a relaparotomy. In addition to these patients, we registered one more case due to a cerebrovascular accident, with a total mortality rate of 12.82%. In the ICG group, the postoperative course was regular in most cases (Table 3). The overall morbidity rate was 20%. There were three complications of grade IIIb according to the Clavien-Dindo classification, two of grade IIIa, one of grade II, and finally two of grade I [10]. Among the complications concerning more specifically the anastomosis, one case of AL occurred (2.5%) in a patient operated on TaTME and affected by chronic ischemic heart disease. It was a small AL (“delimited” according to the classification of Schein [11]), which contributed to an intraoperative malfunction of the circular stapler with only partial suture of the lumen circumference, which was treated and resolved with surgical drainage. There was also one case (2.5%) of stenosis of colorectal anastomosis after resection of the sigma for perforated diverticulitis. The total incidence of complications related to the packaging of the anastomosis in ICG-guided colorectal surgery was therefore 5%. Other complications (one death due to stroke after 30 days with a course complicated by massive hematemesis from duodenal stress ulcer, one reoperation for hemoperitoneum on postoperative day (POD) 2, one reoperation for an unrecognized small bowel lesion due to adhesiolysis on POD 1, one enterocutaneous fistula from radiological drainage of abscess collection on perisplenic hematoma solved spontaneously, and two superficial incisional wound infections (SSI)) did not concern the anastomosis but rather validated excellent perfusion despite episodes of prolonged acute ischemia from postoperative hemorrhage.

**Table 3:** Postoperative complications Values are expressed as n (%). ICG-CRS: indocyanine green-guided colorectal surgery

	ICG-CRS (40 patients)	Control (39 patients)
Overall complications n (%)	8	22
Anastomotic leakage	1 (2.5)	7 (17.9)
Anastomotic stenosis (remote)	1 (2.5)	0 (0)
Abdominal pelvic abscess	1 (2.5)	2 (5.1)
Surgical reintervention	2 (5)	2 (5.1)
Surgical wound infection	2 (5)	5 (12.8)
Ureteral injury	0 (0)	1 (2.6)
Mortality	1 (2.5)	5 (12.8)
Clavien-Dindo classification scale n (%) [10]		
No complications (grade 0)	32 (80)	21 (53.8)
Complications (grade I)	2 (5)	5 (12.8)
Complications (grade II)	1 (2.5)	4 (10.3)
Complications (grade IIIa)	2 (5)	2 (5.1)
Complications (grade IIIb)	3 (7.5)	2 (5.1)
Complications (grade IV)	0 (0)	0 (0)
Mortality (grade V)	1 (2.5)	5 (12.8)

The incidence of AL was compared between the ICG-guided colorectal surgery and control groups using Fisher's exact test due to low expected cell counts. AL occurred in one of 40 patients (2.5%) in the ICG-guided colorectal surgery group and in seven of 39 patients (17.9%) in the control group. The difference was statistically significant (Fisher's exact test, p = 0.029) (Table 4). Although this finding suggests an association between ICG use and a lower incidence of AL, it should be interpreted cautiously because of the retrospective study design, small sample size, and potential confounding arising from baseline and procedural differences between the groups.

**Table 4:** AL incidence in ICG and control groups. Statistical analysis was performed using Fisher's exact test (p=0.029). p<0.05 indicates statistical significance. AL: anastomotic leakage; ICG: indocyanine green; CRS: colorectal surgery

ICG–CRS, n	Control CRS, n	AL ICG-CRS group, n(%)	AL control group, n(%)	Statistical test	P-value
40	39	1 (2,5)	7 (17,9)	Fisher's exact test	0.029

## Discussion

In recent years, we have witnessed important surgical technological advancements (advanced energy devices, electrical staplers with gradual compression and a triple row of stitches, increasingly refined laparoscopic instruments, robotic surgery, and high-resolution imaging systems associated or not with 3D), but the biggest and most dramatic problem of colorectal surgery and consequently also the greatest cause of concern for the surgeon continues to be the packaging and consequently the tightness of the intestinal anastomosis, considering that the percentage of postoperative leakage has remained practically unchanged. In fact, the AL rate varies from 1%-19% depending on the cases and anatomical location: ileocolic (1%-8%); colocolic (2%-3%); ileorectal (3%-7%); colorectal or coloanal (5%-19%) anastomosis. Several factors have been associated with an increased risk of AL: male gender, age, comorbidity, increased American Society of Anesthesiologists (ASA) score [1,3-5], malnutrition, obesity, cigarette smoking, immunosuppression and, in particular, cortisone therapy, alcohol abuse, neoadjuvant radio-chemotherapy, advanced-stage tumors, diverticulitis, low rectal anastomosis, prolonged operating times, blood loss or perioperative transfusions, and intraoperative sepsis conditions [1,3-5]. Above all, an adequate vascular perfusion of the anastomosis is essential for optimal healing and for the prevention of AL [4, 5]. As a result, intraoperative evaluation of intestinal ischemia could significantly reduce its risk during surgery. In the evaluation of intraoperative intestinal perfusion, the use of fluorescence of ICG, a dye already used for other applications since the 1960s, seems to be a method that can potentially reduce the incidence of AL after colorectal surgery. Fluorescence-guided surgery using ICG allows the surgeon to view intestinal microperfusion in real time and is a fast and easy method to implement at the operating table, which can change the surgical resection strategy, as demonstrated by numerous recent studies [7,12-18]

In this retrospective study, the AL rate using guided NIR-ICG imaging is lower (2.5%) than the rate reported in the literature without using this method. If we also consider the case of remote stenosis of the colorectal anastomosis, which required endoscopic dilatative treatment, our results show a relatively low and satisfactory level of complications (7.1%). We report the same percentage (7.5%) for changing surgical strategy (revision of strategy) due to insufficient perfusion as identified by fluorescence of the proximal intestinal stump, which required further resection before the anastomosis. It is very likely that this is the key point of the effectiveness of ICG-based fluorescence, namely a better evaluation of perfusion compared to traditional macroscopic parameters (arterial pulse, bleeding, bowel color, peristalsis, etc.) [18].

Several studies have been published regarding the shifting of the resection line of the intestinal stump [7,14-17]. Wada et al. reported that margin resection was changed in 16% of cases [14], while Jafari et al. reported in their PILLAR II trial a rate of 6.5% of modification of margin resection [7]. Watanabe et al., in their guided ICG rectal resections, brought the section line more proximally towards a more adequate area of fluorescence in 12 cases (5.7%) [16]. Otero-Pineiro et al., during a comparative study on TaTME, reported a variation rate of the proximal resection strategy of 28.7% with an incidence of AL of 2.5% as opposed to 11.3% of the non-ICG group [17]. Morales-Conde et al., in a prospective study, hypothesized that ICG could have a different utility depending on the type of surgery. They analyzed four groups of patients: Group A, right hemicolectomy; Group B, segmental resection of the splenic flexure; Group C, left hemicolectomy; and Group D, anterior rectal resection. Fluorescence with ICG led to significant changes in the section line margin in the left hemicolectomy, followed by rectal resection, and confirmed promise in reducing the AL rate, considering that it occurred in five patients (2.6%): two in Group A (3%), one in Group C (1.2%), and two in Group D (5.7%) [18].

Systematic reviews and meta-analyses also confirm that intraoperative use of fluorescence with ICG is a promising tool for reducing the risk of AL in colorectal surgery. However, the bias inherent in the fact that these are not randomised controlled trials (RCTs) must be taken into consideration when interpreting these data sets. Blanco-Colino et al. analyzed five articles on left colon surgery and reported how the planned level of anastomosis was changed in 7.4% of cases (41 of 555 patients in the ICG-FI group), while showing an overall AL rate of 5.4% [15].

The meta-analysis by Shen et al. reached the same result, even if conducted only on four non-homogeneous, non-randomized studies [19]. It is evident that the considerable heterogeneity and bias related to the mix of the cases operated in this study make it difficult to make an accurate interpretation of the data. As a matter of fact, there are several studies that consider and compare different colorectal interventions. Impellizzeri et al. analyzed left hemicolectomy, sigmoidectomy, and rectal resection interventions, showing a change of strategy in 8.2% of cases with an AL rate of 3% [20]. Ris et al. in a multicenter phase II trial included benign and malignant pathologies operated with different colorectal procedures (right and left hemicolectomy, ileoanal and ileorectal pouches, rectal resections, and Hartmann reversal), and they reported a revision rate of 5.8% with an incidence of AL of 2.4% [21].

In the literature, there are also noteworthy multicenter RCTs. De Nardi et al. evaluated 240 patients operated for left hemicolectomy and rectal resection, divided into two groups with and without ICG. They reported a revision rate of 11% and an AL rate of 5% (for the ICG group) vs 9% without ICG, but it was not statistically significant [22].

Another key multicenter RCT is the IntAct trial. This is the first RCT assessing the use of ICG to prevent AL in rectal resection for cancer. The rates of AL were 36 (10%) in the ICG group and 54 (15%) in the standard care group (p=0.087) [23]. 

The evaluation of the ICG perfusion can be performed according to several steps: before and after the vascular preparation of the proximal intestinal stump, highlighting the fluorescence of the serous side; after the section of the stump on both sides, serous and mucous; and after the packaging of the anastomosis on the serous side.

In the case of a "low" rectal resection (i.e., the distal part of the anorectal canal), the evaluation with ICG of the perfusion of the distal stump is always very difficult to visualize for anatomical reasons on the serous side and is almost exclusively entrusted to the evaluation of the mucous side by endoluminal view [7, 23].

According to a recent experimental study conducted by the group at the IHU Institute in Strasbourg [8], the perfusion of an ischemic segment of the pig colon was analyzed by fluorescence with ICG on the two sides, serous and mucous, and simultaneously the lactate concentration was measured in different areas. The study showed that the average ischemic zone of the mucous side, measured in mm, is significantly wider than the serous one. From this, it can be seen that an evaluation of intestinal perfusion only on the serous side can underestimate the extent of the ischemic area. Knowing this, in our study we also evaluated the mucous side with ICG during the preparation of the proximal stump before the introduction of the stapler and, for the rectum, the evaluation of the same layer by endoanal evaluation after the packaging of the anastomosis. In our study, we evaluated eight out of 28 patients (one rectal anterior resection and five laparoscopic hemicolectomies, one laparoscopic right hemicolectomy and one open right hemicolectomy, four in emergency and four in elective surgery), and none of them experienced AL or other postoperative complications. Further studies will be required to predict the optimal resection margin and the anastomosis site.

Moreover, we believe that the use of fluorescence in surgery is justifiable in relation to costs, considering the significant potential patient benefits. In fact, AL not only increases the risk of mortality and LOS, but it has also been associated with a long-term reduction in tumor-specific survival [1-4].

A similar discourse could be made in the case of emergency surgery. Indeed, with this bowel perfusion assessment, it could be possible to reduce the number of intestinal derivative operations in favor of intestinal resections with primary anastomosis. The World Society of Emergency Surgery (WSES) international position paper concludes that ICG use could provide potential benefits for the prevention of AL in the emergency setting [24]. 

As the most recent data suggest, a net saving in costs is related to lower patient morbidity, reduced hospitalization, and a reduced number of intestinal reconstructions. It is clear that the new technologies must be cost-effective in order for them to be used more widely. Regardless of the mortality and morbidity associated with AL, recent economic assessments suggest that hospital admission costs may increase between €11,900 and €20,300 directly associated with AL complications, and these costs do not include subsequent additional costs related to recanalization, stoma care, and the loss of productivity related to the patient post-discharge [25,26]. The cost for five years of these endoscopic systems is approximately €110,000, while the cost of the ICG for three vials is approximately €130. This shows that if NIR-ICG proved to be truly effective in reducing AL complications, the costs of the technology would be more than adequately covered. The quantitative definition of adequate or inadequate bowel perfusion remains poorly defined, because none of the laparoscopic ICG-fluorescence imaging systems currently available are able to quantify the fluorescent signal. Even if the operator reports a positive visual evaluation, better standardization in the interpretation of the signal would allow for a more objective evaluation. Indeed, the kinetic analysis of the maximum intensity signal could also reveal non-arterial perfusion effects (such as venous return). Automated analysis software programs for more precise and objective quantification have recently been developed, and NIR-ICG systems with quantitative evaluation software are likely to soon enter the market [26-28].

Targeted fluorophores activated by specific cells or microenvironments (perhaps local tissue pH, hypoxia, lactate, or other metabolites) are in development and could allow a more precise identification of tissues than ICG [27-29].

Other experimental techniques have been described to evaluate the blood supply and the integrity of the anastomosis. These include intraoperative endoscopy, pulse oximetry, Doppler ultrasound and Doppler flowmetry, intramucosal pH measurement, and visible light, oxygen spectroscopy and NIR oxygen spectroscopy. None of the techniques mentioned is regularly used, mainly due to its complexity and its high variability of measurements [28-30]. In this regard, Barberio et al. analyzed quantitatively, in an animal model, intestinal perfusion with fluorescence-based enhanced reality (FLER) compared with hyperspectral imaging (HSI) [28].

HSI is another optical imaging technique that combines a spectroscope and a camera, enabling non-contrast, real-time, qualitative, and quantitative tissue analysis based on tissue oxygen saturation (StO₂). The limitation of HSI in the current phase of development is the lack of adequate video speed and the absence of a system for minimally invasive surgery. In the study, HSI produced more accurate results than fluorescence angiography, so that in the future this technology, if made more functional and practical, could be a useful and contrast-free intra-operative tool to quantify intestinal ischemia [27-29].

Our study has several limitations. Most notably, we were unable to perform a multivariable regression analysis to adjust for significant baseline imbalances between the groups, including surgical approach (open vs. minimally invasive) and ERAS protocol adherence. Because these factors are known independent risk factors for AL, a direct causal relationship between ICG use and reduced leak rates cannot be definitively established.

## Conclusions

In conclusion, it is evident that further randomized controlled studies on larger and more homogeneous data are necessary to validate our findings. These results must be interpreted cautiously due to the unadjusted baseline differences between cohorts. Future randomized or propensity-matched studies are required to confirm these findings. In the future, in order to assess more precisely the bowel perfusion with NIR-ICG fluorescence, it also needs a quantitative analysis not only of the serous layer but also of the mucous layer. However, as many studies and trials have already highlighted, the reduction of the incidence of AL, the evidence of a better perfusion of the bowel, and, especially in emergency, the possible reduction of derivative interventions in favor of primary anastomosis are clear. 
